# High proportion of depression and anxiety in younger patients with COPD: a cross-sectional study in primary care in Sweden

**DOI:** 10.1080/02813432.2025.2526667

**Published:** 2025-07-06

**Authors:** Therese Öfverholm, Mikael Hasselgren, Karin Lisspers, Anna Nager, Gabriella Eliason, Maaike Giezeman, Christer Janson, Marta A. Kisiel, Scott Montgomery, Björn Ställberg, Josefin Sundh, Hanna Sandelowsky

**Affiliations:** a NVS, Division of Family Medicine and Primary Care, Karolinska Institutet, Stockholm, Sweden; b Academic Primary Care Centre, Region Stockholm, Sweden; c School of Medical Sciences, Faculty of Medicine and Health, Örebro University, Örebro, Sweden; d Centre for Clinical Research, Region Värmland, Sweden; e Department of Public Health and Caring Sciences, Family Medicine and Preventive Medicine, Uppsala University, Uppsala, Sweden; f Department of Respiratory Medicine, Faculty of Medicine and Health, Örebro University, Örebro, Sweden; g Department of Medical Sciences, Respiratory, Allergy and Sleep Research, Uppsala University, Uppsala, Sweden; h Department of Medical Sciences, Occupational and Environmental Medicine, Uppsala University, Uppsala, Sweden; i Department of Occupational and Environmental Medicine, Akademiska Sjukhuset University Hospital in Uppsala, Sweden; j Clinical Epidemiology and Biostatistics, School of Medical Sciences, Faculty of Medicine and Health, Örebro University, Örebro, Sweden; k Division of Clinical Epidemiology, Department of Medicine, Karolinska Institutet, Stockholm, Sweden; l Department of Epidemiology and Public Health, University College London, London, UK

**Keywords:** COPD, depression, anxiety, primary health care, observational studies, early COPD

## Abstract

**Background and aim:**

Patients with COPD and concurrent depression and/or anxiety are known to have an increased risk of exacerbations, morbidity, mortality, and deteriorated quality of life. Early detection of depression/anxiety may enable early interventions. The aims of this study were to describe the occurrence of depression and anxiety in primary care patients with COPD in Sweden, and to investigate age and gender differences together with other clinical factors associated with this comorbidity.

**Methods:**

A cross-sectional study was performed on a cohort of patients with doctor’s diagnoses of COPD. Patients were randomly selected based on the patients’ contact with 98 primary healthcare centers and 13 hospitals in Sweden in 2014. Information about self-reported depression/anxiety, patient characteristics, symptoms, and comorbidity, were collected using patient self-completion questionnaires. Lung function data were extracted from medical records.

**Results:**

Of the 2245 patients recruited, 23% (*n* = 524) reported depression/anxiety, 29% in women and 16% in men (*p* <0.001). Factors associated with depression/anxiety were being a woman (OR = 2.06 [95% CI 1.56–2.72]), current smoking (1.83 [1.37–2.43]), comorbid asthma (1.77 [1.32–2.37]), dyspnea (the modified Medical Research Council dyspnea scale ≥2 points) (1.58 [1.17–2.13]) and age <65 years (1.57 [1.17–2.10]). The youngest age groups had the highest proportions of patients with depression/anxiety.

**Conclusions:**

Healthcare professionals need to be particularly aware of depression/anxiety in patients with COPD who are younger, women, current smokers, have comorbid asthma, or dyspnea.

## KEY POINTS

- Patients with chronic obstructive pulmonary disease (COPD) and comorbid depression and/or anxiety have an increased risk of exacerbations, comorbidity, and mortality. Early detection may enable early interventions.
- In our study population, nearly one of three women and one of six men with COPD reported having or had depression/anxiety.
- Comorbid depression/anxiety was most common in the youngest age groups.
- Depression/anxiety was associated with younger age, being a woman, current smoking, having comorbid asthma, and high levels of dyspnea.
- An important implication of the study is that primary care professionals should consider the possible occurrence of depression/anxiety in patients with COPD during consultations.

## Introduction

Chronic obstructive pulmonary disease (COPD), along with depression and anxiety, are leading causes of morbidity and mortality in the world [1–3]. In Sweden, the prevalence of COPD is estimated to be 7%, approximately equally distributed between men and women [4]. Depression and anxiety are common in patients with COPD [5]. In Sweden, as well as in the rest of the Nordic countries with similar, government-funded healthcare systems, these conditions are usually managed in primary care, whereas contacts with secondary care are more common in those with severe COPD [6,7].

Having COPD and comorbid depression and/or anxiety (hereafter referred to as “depression/anxiety”) significantly affects patient health. Comorbid depression/anxiety is associated with increased risk of morbidity, exacerbations, and mortality in patients with COPD [8–15]. Moreover, these patients report worse COPD-related health status and health-related quality of life than patients without depression/anxiety [13,16]. Early detection of depression/anxiety may enable early interventions to improve health outcomes and prognosis [11,15].

Previous studies have reported that between 10% and 65% of patients with COPD have comorbid depression/anxiety [17–20]. In a register-based study in Sweden from 2018, depression was found in 24% of the patients with COPD, whereas in a reference population without COPD, the prevalence was 12%. The corresponding prevalence for anxiety disorder was 14% in the COPD population and 7% in the reference population [14]. However, estimating the prevalence of depression/anxiety is challenging as instead of being constant chronic conditions they rather are dynamic conditions that may vary over time. This may contribute to variable prevalence estimations in register-based studies. Patient-reported depression/anxiety could thus be a better way to describe this comorbidity and what characterizes those with symptoms.

As patients with COPD and comorbid depression/anxiety are to be considered a high-risk group, clinicians need to be aware of which of their patients with COPD are at risk of having or developing significant symptoms of these conditions. However, previous studies have presented somewhat contradictory findings. One study reported that the risk of depression/anxiety tended to increase with increasing age and number of comorbidities [21], whereas another study found associations with younger age, female sex, current smoking, and dyspnea [22]. Studies with unselected patients from primary care populations in the Nordic countries are scarce. Moreover, knowledge about the impact of the patient’s age and gender on the development of depression/anxiety in COPD is particularly limited. The aims of this study were to determine the occurrence of depression/anxiety in a cohort of primary care patients with COPD in Sweden, and to investigate gender differences and patient-related factors associated with this comorbidity.

## Materials and methods

### Design and data collection

The present study is part of the PRAXIS study, a longitudinal, observational study about asthma and COPD in central Sweden (2005 – ongoing). Data were cross-sectional and collected from a 2014 PRAXIS cohort of patients with COPD, recruited from eight regions in Sweden (with a total population of 2.5 million inhabitants). Patients were enrolled based on their contacts with 98 randomly selected primary health care centers (PHCC) and 13 hospitals. Eligible patients were 18–75 years old and each of them had a doctor’s diagnosis of COPD (ICD-10 code J44) registered in the medical records between January 2007 and December 2010. Between 22 and 45 patients were randomly selected from each site. Postal invitations including patient questionnaires and written informed consent forms were sent to 3877 patients. Reminders were sent twice to non-responders. Patients were asked for written consent to review their medical records.

Patients who agreed to participate replied to the invitation by completing a patient questionnaire about current or previous depression/anxiety, age, gender, weight/height, education, physical activity, smoking habits, exacerbation history, age of onset of COPD symptoms, comorbidity, and current symptoms of COPD.

### Definition of depression/anxiety

As depression and anxiety share several features regarding symptoms, diagnostics, and treatment, we chose to study the diagnoses together. As previous studies have shown that the risk of negative COPD outcomes, such as exacerbations and mortality, are shared by patients with anxiety and depression, the conditions were combined into one group of depression/anxiety [9,11,23]. To define this group we used information on self-reported depression/anxiety from the patient questionnaire. Those who answered “yes” to the following question in the patient questionnaire: “Do you have/have you had depression/anxiety?” were included.

### Comorbidity

Comorbidity was defined as self-reported comorbidity from the patient questionnaire. The chosen comorbidities are known to be common in patients with COPD [14,23]. Patients were asked “do you have/have you had any of these following conditions?” and the patients answered yes/no to each condition; asthma, chronic bronchitis, allergies, heart disease, diabetes, stroke, hypertension, sleep apnea, rheumatic disease, cancer.

### Variables

The variable patient age was dichotomized as <65 and ≥65 years, which is the official age of retirement in Sweden. Additionally, patient age was grouped in intervals of 10 years, to enable descriptions of age distributions regarding reported depression/anxiety. The body mass index (BMI) was calculated as kg/m^2^. Underweight was defined as BMI <18.5, normal weight as BMI ≥18.5 and <25, overweight as BMI ≥25 and <30, and obesity as BMI ≥30 [24]. Education was dichotomized as low and high level of education, and high educational level was defined as those who had completed >2 years of education after 9 years of compulsory school. Physical activity was dichotomized as being mostly inactive and active ≥4 h/week. Smoking status was dichotomized as never/former and current smoker. An exacerbation was defined as worsening of respiratory symptoms in need of an emergency visit to primary or secondary care and/or need for an oral course of steroids and/or antibiotics, during the previous six months. The exacerbation variable was dichotomized as no exacerbation and ≥1 exacerbation previous six months. The variable symptom onset age was dichotomized as ≤60 and >60 years, based on the possible choices in the patient questionnaire (<30, 30–50, 51–60, 61–70, and >70 years). The cut-off at ≤60 and >60 years made the two groups as equal in size as possible.

The modified Medical Research Council dyspnea scale (mMRC) [25] was used to assess activity-related breathlessness. The mMRC scored from 0 (breathless only with strenuous exercise) to 4 (too breathless to leave the house or breathless when getting dressed). The scores were dichotomized with the cut-off mMRC ≥2, which defines clinically significant breathlessness in everyday life [26]. The COPD Assessment Test (CAT) [27] was used to assess the impact of COPD symptoms on patients’ health status and daily life. The eight questions referred to symptoms of cough, sputum, chest tightness, breathlessness, limitations in activities, confidence in leaving the house, sleep, and energy. The scoring scale ranged between 0 and 5 for each item, and the total score ranged from 0 to 40. The higher the score, the greater the impact of COPD on the patient’s health status. The Clinical COPD Questionnaire (CCQ) was used to assess health-related quality of life [13]. It consisted of 10 items, divided into three domains: symptoms, functional state, and mental state. The scoring scale of each item ranged from 0 (best) to 6 (worst). The final measure is the mean value of the 10 items [28]. The higher the score, the greater the negative impact on health-related quality of life [13].

Lung function data were collected from medical records and included the forced expiratory volume in one second (FEV_1_) and the forced vital capacity (FVC). Data from the last performed spirometry between 2004 and 2014 were used. The severity of the airflow limitation was classified according to the Global Initiative for Chronic Obstructive Pulmonary Disease [1] as stage 1 (mild) FEV_1_ ≥80% of predicted, stage 2 (moderate) 50%≤ FEV_1_ <80% of predicted, stage 3 (severe) 30%≤ FEV_1_ <50% of predicted, stage 4 (very severe) FEV_1_ <30% of predicted.

### Statistical analysis

Summary statistics such as means, proportions, and measures of dispersion were assessed using standard parametric methods. The difference between COPD patients with and without depression/anxiety, and between men and women, were assessed by Chi^2^ test and *T*-test. Univariate logistic regression analyses were used to examine possible associations between depression/anxiety and age, gender, BMI, education, physical activity, smoking habits, exacerbation history, mMRC, and the following comorbidity: asthma, chronic bronchitis, allergies, heart disease, diabetes, stroke, hypertension, sleep apnea, rheumatic disease, and cancer. Then, variables with statistically significant associations in the univariate analyses which were considered clinically relevant were used in a multivariable logistic regression model with adjustment for age, gender, physical activity, education, BMI, exacerbation previous six months, and lung function (FEV_1_% of predicted) which also provided odds ratios (OR) and their 95% confidence intervals (CI). To address the problem with multicollinearity, stratification and interaction analyses were performed for both gender and age to evaluate whether the factors associated with depression/anxiety differed between the genders and the different age groups of <65 and ≥65 years. *p* Values <0.05, and 95% confidence interval indicated statistical significance. The statistical analysis was performed using SPSS software Version 28.0.1.1 (IBM Corp. Armonk, NY).

## Results

A total of 2245 patients (response rate 58%) replied to the questionnaire. Of the 2245 patients, 1809 (81%) were recruited based on their COPD diagnoses in primary care records and 434 (19%) in secondary care records. Of the secondary care recruited, 31% reported ≥1 emergency visit in primary care over the last six months.

**Figure 1.**
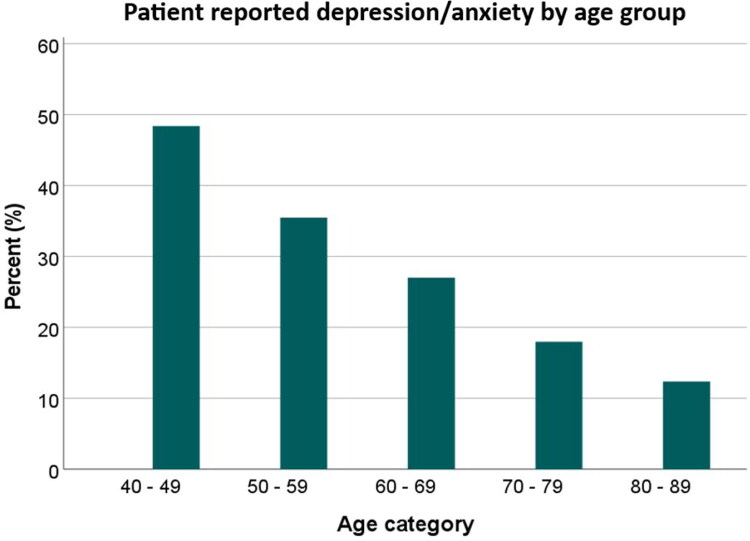
Reported depression/anxiety across patient age groups. Data is presented as a percentage of the total number of individuals in each respective age category. Categories with <10 individuals are excluded from the picture.

Out of the study population, 524 (23%) reported having current or previous depression/anxiety, 29% in women and 16% in men (*p* <0.001). The patient characteristics are summarized in Table 1. The patients with depression/anxiety were generally younger than those without depression/anxiety. The proportions of reported depression/anxiety were the highest in younger patients; of the 40–49-year-olds 48.8% and of the 50–59-year-olds 35.5% reported depression/anxiety, whereas 12.4% of the patients >80 years did so (Figure 1). Patients with depression/anxiety had an onset of COPD symptoms earlier in life than those without depression/anxiety. They were also more often overweight, physically inactive, and current smokers. A recent (within six months) COPD exacerbation, symptoms of clinically significant dyspnea (mMRC ≥2), and worse COPD-related health status and health-related quality of life were more common in patients with depression/anxiety than in those without. Comorbid asthma, allergy, chronic bronchitis, and rheumatic disease were more common in patients with depression/anxiety, whereas other co-existing chronic conditions such as diabetes, heart disease, stroke, hypertension, or cancer, were not more common in these patients. No significant differences in lung function measures, GOLD stage, or educational levels were found between patients with and without depression/anxiety.

**Table 1.** Patient characteristics of the total cohort and of patients with/without depression/anxiety.

Patient characteristics	Total n= 2245	No depression/ anxiety *n* = 1721	Have/had depression/anxiety *n* = 524	*p*-value
*Patient age, mean (SD)*	68.6 (7.4)	69.3 (7.2)	66.5 (7.7)	<0.001
*Patient age,* n *(%)*				
<65	549 (24.5)	371 (21.6)	178 (34.0)	<0.001
≥65	1696 (75.5)	1350 (78.4)	346 (66.0)	
*Patient age categories,* n *(%)*				
<40	1 (0.0)	1 (100.0)	0	<0.001
40–49	31 (1.4)	16 (51.6)	15 (48.4)	
50–59	217 (9.7)	140 (64.5)	77 (35.5)	
60–69	892 (39.7)	651 (73.0)	241 (27.0)	
70–79	990 (44.1)	812 (82.0)	178 (18.0)	
80–89	105 (4.7)	92 (87.6)	13 (12.4)	
≥90	9 (0.4)	9 (100.0)	0	
*Gender,* n *(%)*				
Women	1269 (56.5)	899 (52.2)	370 (70.6)	<0.001
Men	976 (43.5)	822 (47.8)	154 (29.4)	
*BMI* ^*1*^ *^,^ mean (SD)*	26.5 (5.5)	26.3 (5.3)	26.9 (6.1)	0.035
*BMI*^*1*^ *categories,* n *(%)*				
<18.5	110 (4.9)	79 (4.6)	31 (5.9)	0.023
18.5–24.9	822 (36.6)	647 (37.6)	175 (33.4)	
25–29.9	731 (32.6)	572 (33.2)	159 (30.3)	
≥30	582 (25.9)	423 (24.6)	159 (30.3)	
*Educational level,* n *(%)*				
Lower	1674 (77.0)	1281 (77.0)	393 (76.9)	0.995
Higher	500 (23.0)	382 (23.0)	118 (23.1)	
*Physical activity,* n *(%)*				
Mostly inactive	536 (33.0)	381 (31.2)	155 (38.6)	0.006
Active ≥4h/week	1088 (67.0)	841 (68.8)	247 (61.4)	
*Smoking status,* n *(%)*				
Current smoker	702 (32.1)	482 (28.7)	220 (43.1)	<0.001
*Smoking, pack years, mean (SD)*	31.1 (18.9)	30.7 (18.9)	32.3 (18.8)	0.124
*Exacerbation previous six months,* n *(%)*	757 (34.2)	558 (32.9)	199 (38.5)	0.019
*Symptom onset age,* n *(%)*				
<60	1329 (62.6)	964 (59.4)	365 (73.0)	<0.001
≥60	795 (37.4)	660 (40.6)	135 (27.0)	
*Asthma,* n *(%)*	563 (25.1)	391 (22.7)	172 (32.8)	<0.001
*Chronic bronchitis,* n *(%)*	138 (6.1)	95 (5.5)	43 (8.2)	0.025
*Allergies,* n *(%)*	639 (37.7)	461 (36.3)	178 (41.8)	0.042
*Heart disease,* n *(%)*	482 (21.5)	378 (22.0)	104 (19.8)	0.302
*Diabetes,* n *(%)*	343 (15.3)	261 (15.2)	82 (15.6)	0.788
*Stroke,* n *(%)*	173 (7.7)	126 (7.3)	47 (9.0)	0.215
*Hypertension,* n *(%)*	1052 (46.9)	826 (48.9)	226 (43.1)	0.051
*Sleep apnea,* n *(%)*	248 (11.0)	181 (10.5)	67 (12.8)	0.147
*Rheumatic disease,* n *(%)*	229 (10.2)	163 (9.5)	66 (12.6)	0.039
*Cancer,* n *(%)*	299 (13.3)	234 (13.6)	65 (12.4)	0.479
*mMRC*^*2*^, n *(%)*				
0–1	1073 (49.7)	862 (52.1)	211 (41.9)	<0.001
2–4	1084 (50.3)	792 (47.9)	293 (58.1)	
*CAT*^*3*^, *mean (SD)*	16.0 (8.5)	15.2 (8.3)	18.6 (8.8)	<0.001
*CCQ*^*4*^ *mean (SD)*	1.97 (1.3)	1.84 (1.2)	2.38 (1.4)	<0.001
*FEV_1_*^*5*^ *% of predicted, mean (SD)*	57.0 (18.6)	56.8 (18.5)	57.8 (18.6)	0.296
*GOLD*^*6*^ *stage,* n *(%)*				
1	197 (10.2)	147 (10.0)	50 (11.0)	0.055
2	1060 (55.1)	791 (53.8)	269 (59.1)	
3	537 (27.9)	433 (29.5)	104 (22.9)	
4	130 (6.8)	98 (6.7)	130 (6.8)	

**Abbreviations**.

^1^BMI: Body mass index.

^2^mMRC: Modified Medical Research Council Dyspnea Scale.

^3^CAT: COPD Assessment Test.

^4^CCQ: Clinical Chronic Obstructive Pulmonary Disease Questionnaire.

^5^FEV_1_: Forced expiratory volume in one second.

^6^GOLD: Global Initiative for Chronic Obstructive Lung Disease.

Gender differences in patients with depression/anxiety are shown in Table 2. Of the 524 patients with depression/anxiety, 370 (70.6%) were women. Men had smoked more (a higher number of packyears) than women, but otherwise the characteristics did not differ between the genders. Stroke and sleep apnea were more common in men, and rheumatic disease was more common in women.

**Table 2.** Gender differences in characteristics of patients who have/have had depression/anxiety, *n* = 524.

Patient Characteristics	Women *n* = 370	Men *n* = 154	*p*-value
*Patient age, mean (SD)*	66.7 (7.6)	65.8 (7.9)	0.243
*Patient age,* n *(%)*			
<65	120 (32.4)	58 (37.7)	0.250
≥65	250 (67.6)	96 (62.3)	
*Patient age categories,* n *(%)*			
<40	0 (0.0)	0 (0.0)	0.698
40–49	9 (2.4)	6 (3.9)	
50–59	53 (14.3)	24 (15.6)	
60–69	170 (45.9)	71 (46.1)	
70–79	127 (34.3)	51 (33.1)	
80-89	11 (3.0)	12 (1.3)	
≥90	0 (0.0)	0 (0.0)	
*BMI*^*1*^, *mean (SD)*	26.9 (6.1)	26.9 (6.0)	0.970
*BMI*^*1*^ *categories,* n *(%)*			
<18.5	23 (6.2)	8 (5.2)	0.238
18.5–24.9	117 (31.6)	58 (37.7)	
25–29.5	112 (30.3)	47 (30.5)	
≥ 30	118 (31.9)	41 (26.6)	
*Educational level,* n *(%)*			
Lower	280 (77.8)	113 (74.8)	0.471
Higher	80 (22.2)	38 (25.2)	
*Physical activity,* n *(%)*			
Mostly inactive	109 (39.4)	46 (36.8)	0.627
Active ≥4h/week	168 (60.6)	79 (63.2)	
*Smoking status,* n *(%)*			
Current smoker	162 (45.3)	58 (38.2)	0.139
*Smoking, pack years, mean (SD)*	30.5 (17.3)	36.7 (21.6)	0.002
*Exacerbation previous six months,* n *(%)*	138 (37.7)	61 (40.4)	0.567
*Symptom onset age*, n *(%)*			
<60	260 (73.2)	105 (72.4)	0.850
≥60	95 (26.8)	40 (27.6)	
*Asthma,* n *(%)*	129 (34.9)	43 (27.9)	0.123
*Chronic bronchitis,* n *(%)*	29 (7.8)	14 (9.1)	0.634
*Allergies,* n *(%)*	127 (42.9)	51 (39.2)	0.479
*Heart disease,* n *(%)*	71 (19.2)	33 (21.4)	0.558
*Diabetes,* n *(%)*	56 (15.1)	26 (16.9)	0.616
*Stroke,* n *(%)*	21 (5.7)	6 (16.9)	<0.001
*Hypertension,* n *(%)*	160 (43.2)	66 (42.9)	0.935
*Sleep apnea,* n *(%)*	37 (10.0)	30 (19.5)	0.003
*Rheumatic disease,* n *(%)*	57 (15.4)	9 (5.8)	0.003
*Cancer,* n *(%)*	49 (13.2)	16 (10.4)	0.367
*mMRC*^*2*^, n *(%)*			
0–1	147 (41.3)	64 (43.2)	0.686
2–4	209 (58.7)	84 (56.8)	
*CAT*^*3*^, *mean (SD)*	18.4 (8.7)	19.0 (9.2)	0.510
*CCQ*^4^, *mean (SD)*	2.4 (1.3)	2.5 (1.4)	0.451
*FEV_1_*^5^ *% of predicted, mean (SD)*	58.1 (18.1)	57.1 (19.8)	0.589
*GOLD*^*6*^ *stage,* n *(%)*			
1	33 (10.3)	17 (12.7)	0.593
2	192 (59.8)	77 (57.5)	
3	76 (23.7)	28 (20.9)	
4	20 (6.2)	12 (9.0)	

**Abbreviations**.

^1^BMI: Body mass index.

^2^mMRC: Modified Medical Research Council Dyspnea Scale.

^3^CAT: COPD Assessment Test.

^4^CCQ: Clinical Chronic Obstructive Pulmonary Disease Questionnaire.

^5^FEV_1:_ Forced expiratory volume in one second.

^6^GOLD: Global Initiative for Chronic Obstructive Lung Disease.

**Figure 2.**
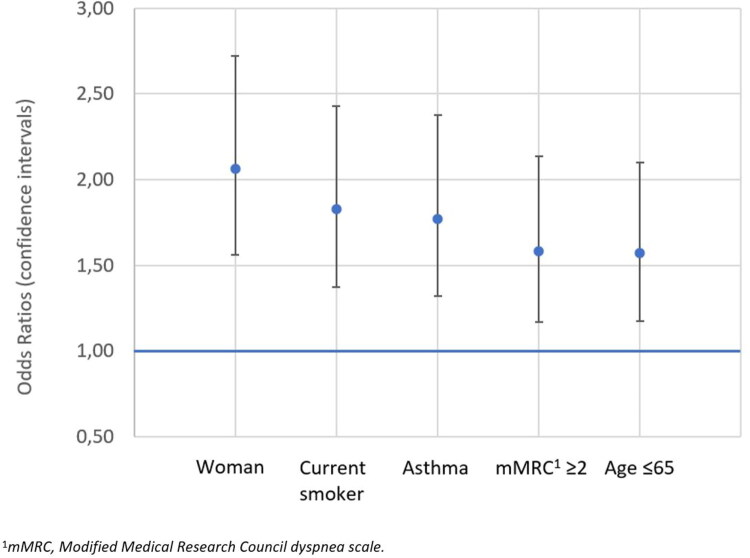
Associations of depression/anxiety with patient-related factors estimated by multivariable logistic regression analysis, adjusted for physical activity, education, BMI, exacerbation previous six months, and lung function (FEV_1_% of predicted).

In the multivariable logistic regression analysis of factors associated with comorbid depression/anxiety, significant associations were shown between depression/anxiety and being a woman (OR = 2.06 [95% CI 1.56−2.72]), current smoking (1.83 [1.37−2.43]), comorbid asthma (1.77 [1.32−2.37]), mMRC score ≥2 points (1.58 [1.17−2.13]) and age <65 years (1.57 [1.17−2.10]) (Figure 2). BMI, education, physical activity, exacerbation history, or lung function (FEV_1_% of predicted) were not significantly associated with depression/anxiety (Table 3).

**Table 3.** Multivariable logistic regression analysis, dependent variable: to have/have had depression/anxiety.

Factor	Unadjusted OR^*1*^	95% CI^*2*^	*p-*value	Adjusted OR^*1*^	95% CI^*2*^	*p-*value
Woman	2.20	1.78–2.77	<0.001	2.06	1.56–2.72	<0.001
Current smoking	1.88	1.53–2.31	<0.001	1.83	1.37–2.43	<0.001
Comorbid asthma	1.66	1.34–2.10	<0.001	1.77	1.32–2.37	<0.001
mMRC^*3*^ ≥2	1.51	1.24–1.85	<0.001	1.58	1.17–2.13	0.003
Patient age <65 years	1.87	1.51–2.32	<0.001	1.57	1.17–2.10	0.002
Physical activity, mostly inactive	1.39	1.10–1.75	0.006	1.29	0.96–1.73	0.098
Education	0.99	0.79–1.26	0.955	0.79	0.58–1.09	0.154
BMI^*4*^	1.02	1.00–1.04	0.035	1.01	0.99–1.03	0.290
Exacerbation previous six months	1.28	1.04–1.57	0.019	0.97	0.72–1.30	0.829
FEV_1_^5^ % of predicted	1.00	0.99–1.01	0.296	1.01	0.99–1.01	0.235

All factors adjusted for each other in the analysis.

**Abbreviations**.

^1^OR: Odds Ratio.

^2^CI: Confidence Interval.

^3^mMRC: Modified Medical Research Council Dyspnea Scale.

^4^BMI: Body mass index.

^5^FEV_1_: Forced expiratory volume in one second.

When stratified by gender and by age <65 and ≥65 years, differences in the associations between depression/anxiety and some independent variables were observed across the stratification groups. However, interaction terms included in the overall model were not statistically significant.

## Discussion

In this study of 2245 patients with COPD in Sweden, we found that 23% reported current or previous depression/anxiety, corresponding to 29% of the women and 16% of the men. Men had smoked more than women, but otherwise there were no differences in characteristics between men and women with COPD and concomitant depression/anxiety, and the proportions of patients with reported depression/anxiety were the highest in the youngest age categories. Moreover, we found that depression/anxiety was associated with being a woman, current smoking, comorbid asthma, symptoms of dyspnea (mMRC score ≥2) and age <65 years.

### Our findings in relation to previous work

Depression/anxiety was found to be common in our study population, as nearly one out of four reported current or previous episodes of depression/anxiety [14,29]. In the general population of Sweden, the occurrence of self-reported severe psychological distress, including symptoms of anxiety and depression, has been reported to be 12% in women and 10% in men [30]. The proportion of depression/anxiety we identified in our cohort aligns with previous findings from a register-based population study from Sweden where 24% of the patients with COPD had a registered diagnosis of depression, compared to 12% in matched controls without COPD [14]. Moreover, our findings are consistent with the ECLIPSE study that found an occurrence of depression in 26% of patients with COPD, based on large patient-reported data. However, the study population in ECLIPSE may have differed from ours as the subjects were included from inpatient clinics from 12 European countries, and in general had more severe COPD than those included in our study [22,31]. Our study population was representative of a primary care population in a Nordic healthcare system, in which primary care has the main responsibility of COPD care, occasionally in co-operation with secondary care [14,32].

We found a significant association between depression/anxiety and younger age in patients with COPD, thus aligning with the results of the ECLIPSE study [22]. On the other hand, previous studies have found associations between depression/anxiety and increasing age [21] and time since COPD diagnosis [5]. Possible explanations for these discrepancies in different study results could be that our definition of depression/anxiety was based on patient-reported data rather than solely on doctors’ diagnoses, and that our findings are specific to Swedish primary care populations.

In our study population, depression/anxiety was more than twice as common in women as in men which was anticipated, as these conditions are generally more common in women [14,32,33]. However, how men and women with COPD and depression/anxiety may differ, has been scarcely studied. While a 2006 Italian study reported that dyspnea was more strongly associated with depression in women than in men with COPD [34] we could not confirm this finding in our study population.

In line with other studies, we found that current smoking was associated with comorbid depression/anxiety in patients with COPD [35]. Depression has been reported to hamper smoking cessation [36] and patients living in socioeconomically deprived areas are more likely to smoke [37]. Therefore, primary care professionals need to be particularly aware that patients in socioeconomically deprived areas have an increased risk of developing multimorbidity, insufficient disease control, and depression/anxiety [38].

In the present study, we found a positive association between comorbid asthma and depression/anxiety. Previous studies of the relationship between asthma and depression/anxiety in COPD have reported contradicting results [39,40], but to our knowledge, this has not been previously studied in the Nordic countries.

### Increased risk of depression/anxiety in younger patients with COPD

The mechanisms behind the overall increased risk of depression/anxiety in patients with COPD are not completely understood. According to a bidirectional explanatory model [41] depression/anxiety have adverse effects on COPD outcomes through reduced physical activity and adherence to treatment [42], and vice versa: COPD may increase the risk of developing depression/anxiety through reduced physical capacity, airflow limitation, and dyspnea. In line with this, we found physical inactivity and exacerbations to be more common in patients with depression/anxiety. However, the development of depression in COPD is multifactorial, where genetic, environmental, systemic inflammatory, and lifestyle factors (such as smoking) may contribute together with the load of chronic illness. The increased burden of several stressors affecting each other, creating high allostatic load, may lead to the development and maintenance of mental comorbidity in these patients [35,43,44].

We speculate that a possible explanation to the higher prevalence of depression/anxiety in younger patients with COPD may be related to higher demands on functionality and health in general in younger people. Additionally, depression/anxiety in older people may often be hidden behind somatic symptoms, either because of somatization of the depression/anxiety disorder or because of accentuation of symptoms of COPD [45]. This may have led to some underreporting of depression/anxiety in the older patients in our study. However, younger female COPD patients may have found it more acceptable to report symptoms of depression/anxiety, which may have affected our results.

### Strengths and limitations

The large recruitment of real-world primary care patients was a major strength of this study. The gender distribution was fairly equal in the main study population, and thus well represented the general COPD population in Sweden [4]. Patients recruited from secondary care reported frequent emergency visits in primary care, so it is reasonable to assume that all recruited patients were representative of patients commonly seen in primary care. As primary care COPD populations are similar in all Nordic countries, the results are likely to be generalizable in the Nordics.

Another important strength of our study was that we were able to identify patients who themselves expressed current or previous depression/anxiety, rather than to rely on register-based diagnoses, which distinguishes our study from other studies on the topic. Our study describes the patients that general practitioners meet every day in their clinical work. As depression and anxiety are known to be underdiagnosed in the public in general [46] and in patients with COPD [18,47], self-reported depression/anxiety data enabled us to study previously undiagnosed individuals. By describing the proportion of patients with symptoms, we highlight the potentially undiagnosed patients who are at risk of negative outcomes. However, relying only on self-reported symptoms may have led to an overestimation of the diagnoses due to possible unmet established diagnostic criteria. On the other hand, underestimation may have occurred due to recall bias, especially when the symptoms were in remission. Using a validated screening tool for depression/anxiety, such as Patient Health Questionnaire 4 (PHQ4) [48] or Hospital Anxiety and ‘Depression Scale (HADS) [49], to define the diagnoses would have increased the comparability of our results with other studies, and thus increased the external validity. The use of HADS would also have made it possible to distinguish between, and assess the occurrence of, the diagnoses of depression and anxiety, and more closely describe subtypes of each of these conditions.

Although data were collected 11 years ago, more recent studies have reported that the occurrence of depression and anxiety remains high in patients with COPD, and the underdiagnosis thus remains common [47,50].

### Implications and need for further research

Primary care professionals should take the possible occurrence of depression/anxiety into consideration during consultations, even when patients actively do not bring it up. The use of validated screening tools in clinical practice would facilitate the detection of depression/anxiety in patients with COPD. Additionally, it is important to optimize the treatment of both depression/anxiety and COPD. Whether this leads to a reduced risk of exacerbations, improved COPD control, and increased physical activity, is an important subject of future research.

## Conclusion

In primary care, nearly one of three women and one of six men with COPD, particularly the younger patients, may suffer from depression/anxiety. Men and women with COPD and depression/anxiety have similar clinical characteristics. General practitioners should be aware of symptoms of depression and anxiety in COPD patients, specifically in younger patients, women, current smokers, and patients with comorbid asthma or symptoms of dyspnea.

## Data Availability

The dataset analyzed in the present study contains confidential information and is not publicly available due to ethical restrictions.
